# Using smart devices for prenatal care: Assessing the willingness among women with pregnancy-related anxiety

**DOI:** 10.1177/20552076251406652

**Published:** 2026-01-27

**Authors:** Stefanie Altmannshofer, Filip Weidenthaler, Adriana Titzmann, Constanza A Pontones, Nina Danzberger, Katharina M Jaeger, Michael Nissen, Heike Leutheuser, Bjoern M Eskofier, Peter A Fasching, Matthias W Beckmann, Hanna Huebner

**Affiliations:** 1 Department of Gynecology and Obstetrics, 27168 Universitätsklinikum Erlangen, Friedrich-Alexander-Universität Erlangen-Nürnberg (FAU), Erlangen, Germany; 2 Machine Learning and Data Analytics Lab, 9171 Friedrich-Alexander-Universität Erlangen-Nürnberg (FAU), Erlangen, Germany; 3 Ambient Assisted Living & Medical Assistance Systems, Department of Computer Science, 26523 University of Bayreuth, Bayreuth, Germany

**Keywords:** Pregnancy-related anxiety, PRAQ-R2, prenatal care, mHealth, wearables

## Abstract

**Background:**

Wearables and smart devices could complement face-to-face prenatal care appointments by monitoring pregnant women's health, especially since pregnancy may be a vulnerable time when mental health issues and pregnancy-related anxiety may arise.

**Aim:**

The aim of this study was to analyze the extent whether the willingness of women to use smart devices for pregnancy care monitoring differs between those with pregnancy-related anxiety and those without.

**Methods:**

A survey was conducted to ascertain participant's general characteristics, attitudes toward smart devices, the willingness to use them, and the level of pregnancy-related anxiety (PRAQ-R2). Associations between the willingness and pregnancy-related anxiety parameters were analyzed.

**Results:**

Completed questionnaires from 210 women were included in the analysis. A significant difference between women showing high and low levels of pregnancy-related anxiety was observed in terms of their willingness to use a smartwatch (median score 4.00; interquartile range (IQR) 4.00–5.00 vs. 4.00; IQR 3.00–4.00; *P* = .02), smart contraction counter (median score 4.00; IQR 3.00–5.00 vs. 3.00; IQR 3.00–4.00; *P* = .02), smart blood pressure monitor (median score 5.00; IQR 3.00–5.00 vs. 4.00; IQR 3.00–4.00; *P* = .003) or sleep tracker (median score 4.00; IQR 3.00–5.00 vs. 3.00; IQR 2.00–4.00; *P* = .007). Overall, anxious women showed significantly higher willingness to use smart devices in the context of prenatal care.

**Conclusions:**

The data suggest that pregnant women are receptive to using smart devices to enhance their prenatal care, particularly those experiencing higher levels of anxiety. This study serves as an initial step in evaluating attitudes toward these devices. As a follow-up, it is recommended that acceptance and feasibility studies are conducted alongside the further development of existing and new pregnancy-specific wearables.

## Introduction

A woman's lifestyle, behavior, and physical activities during pregnancy can affect her health and that of the fetus^1^; ongoing monitoring may become necessary if risks or complications arise that have the potential to result in adverse outcomes such as miscarriage, preterm birth, stillbirth, or low birth weight.^2^ The typical course of prenatal care in Germany entails 10 to 12 face-to-face visits to a healthcare provider, encompassing the provision of information and education to the patient, ultrasound scans, examination of basic vital parameters such as heart rate, blood pressure and glucose levels, tests for various infections, and monitoring of high-risk pregnancies.^3^

Recent advances in technology have enabled the development of numerous devices and sensors that provide people with the means to remotely monitor and track health conditions in their day-to-day lives. Wearable sensors, mobile health (mHealth) technologies, mobile apps, and other wireless devices, such as smartwatches, have opened up new possibilities for the continuous monitoring of behavioral and physiological parameters.^4^ Recent research has found health apps to be effective in helping prevent cardiovascular disease,^5^ improving their nutrition,^5^ and in the treatment of depression.^6^ Other work suggests that patients are able, to a significant extent, to carry out health monitoring via apps autonomously, and indicates the utility of self-tracked data for predictions and simulations.^7^ This research highlights the potential of remote monitoring to reduce in-clinic visits while improving health monitoring.^7^ The use of telemedicine technologies may help alleviate the current shortage of physicians by enabling medical check-ups to be conducted in the patient's home environment, thereby optimizing physicians’ time and resources.^8^

Pregnancy is one area in which telemedicine has notable potential. Ultrasound scans, the monitoring of uterine contractions and tracking of fetal heartbeat as well as monitoring of vital signs like blood pressure, body temperature, heart rate or body weight changes are standard components of routine prenatal care aiming to detect high-risk pregnancies as early as possible in order to enable the best possible treatment. Notwithstanding the fact that trained medical professionals usually conduct these examinations, research suggests it may be possible for patients to carry out monitoring of these parameters, at least in part, at home. Self-guided ultrasound examinations, for instance, elicited a broadly positive response among women^9^ and automated monitoring of uterine contractions via surface electrodes attached to the skin appears to be suitable for telemonitoring in pregnancy.^10^ The use of smart devices for monitoring of additional parameters during pregnancy may also offer significant benefits. Various lifestyle factors, such as physical activity, sleep habits, stress, diet, and weight management are known to influence pregnancy outcome^2^; as an example, physical exercise adapted for pregnancy may cut the risk of preeclampsia, prevent gestational diabetes, and reduce the occurrence of postpartum musculoskeletal issues.^2^ The measurement of blood pressure is of particular significance due to physiological changes that affect vessel wall compliance, leading to a risk of hypertensive disorders such as preeclampsia. Research has found self-monitoring of blood pressure to meet with positive attitudes among patients and healthcare providers alike, with ease of use, convenience, and reduced anxiety cited as particular benefits.^11–13^ In addition to well-known parameters that are already part of routine care, novel physiological information could be obtained from wearable devices. As so, sleep quality and quantity might be parameters relevant for future prenatal monitoring. Altered sleep patterns of pregnant women may result in disturbed sleep and other disorders which in turn may have a negative impact on maternal and child health, by, for example, increasing the risk of preterm birth or intrauterine growth restriction.^14^ To date, however, the monitoring and management of sleep patterns are not an integral part of standard prenatal care.^15^ The potential of smart devices in this context is noteworthy, given their capacity to facilitate the monitoring of sleep patterns, physical exertion, and vital signs, including heart rate. The real-time data collected by wearables could therefore supplement traditional in-person prenatal visits, providing additional information and a more comprehensive view of pregnancy health that helps healthcare providers tailor careplans and give timely advice. Further, embracing smart devices for prenatal care presents opportunities also for underdeveloped nations and rural areas, where growing healthcare provider shortages are challenging: Smart devices can substantially improve prenatal monitoring where access to traditional healthcare facilities is limited or unreliable. In these regions, smart devices for prenatal care have been proposed to remotely transmit data to healthcare providers, overcoming geographic and resource barriers and facilitating timely interventions.^12,16,17^ However, there are also some pitfalls that must be considered when recommending wearable devices: constant monitoring might represent an additional source of anxiety and stress, reinforcing reassurance-seeking behavior and potentially worsening symptoms of pathological anxiety.^18^ Further, wearable data often struggle with data quality and may produce biased or unbalanced estimates, which can result from factors such as user error, poor sensor placement, or device limitations.^19^ In addition, not all individuals benefit equally from wearables since unequal access, often dictated by socioeconomic status or digital literacy, may potentially reinforce existing inequalities in health outcomes, especially in low-income countries and underserved regions.^19^ Challenges such as low education, poor internet connection, and insufficient infrastructure may hinder the effective use of smart wearable devices, especially in rural areas and underdeveloped nations.^17,19^

Research on maternal mental health has now widened its focus from the postpartum stage alone to the prenatal period. Several studies in this context have evidenced the negative impact of prenatal maternal anxiety on pregnancy and birth outcomes.^20^ The diagnosis of an anxiety disorder is associated with poor maternal health, adverse birth outcomes, and negative behavioral and biological development in children across their lifespan.^21^ Pregnancy-related anxiety (PrA) is distinct from general anxiety and anxiety disorders.^22^ It is defined as fear, worry or apprehension associated with pregnancy, childbirth, the health of the fetus or baby, and pregnancy-related psychosocial or financial problems, including a woman's fears and concerns about her physical appearance during pregnancy and her ability to meet her expectations of herself as a parent.^23^

Despite the increasing potential of integrating wearable or other sensor technologies into prenatal care, little is known about how pregnant women perceive such devices and how psychological factors such as PrA influence their acceptance and intended use. By addressing these gaps, digital solutions for prenatal care can be tailored to the women's emotional needs. To investigate these questions, we conducted a survey among pregnant women. The specific objectives of this study are therefore as follows:

- To evaluate pregnant women's perceptions and attitudes toward the use of smart sensor devices in prenatal care.
- To analyze whether pregnant women experiencing anxiety demonstrate different levels of willingness to use smart devices for prenatal care compared to those without anxiety.
- To understand these potential differences in order to inform the design and tailoring of digital health solutions, ensuring they better meet the unique needs of pregnant women, particularly accounting for the influence of anxiety on health behavior and decision-making.

These objectives aim to provide insight into how anxiety impacts acceptance of prenatal smart technology, guiding improved implementation and patient-centered approaches in digital prenatal care.

## Methods

### Study design and population

The open, prospective and single-center study SMART Start aimed to evaluate the acceptance and feasibility of various smart devices for home-based health monitoring during pregnancy. These devices included smartwatches, smart contraction counters, smart blood pressure monitors (BPMs), and smart sleep trackers. Within this study, a survey was conducted, with the primary objective of analyzing pregnant women's willingness to use these smart devices as part of their prenatal care routine. Women from week 9 of pregnancy onward were included. Racial, ethnic, or national background data was not collected. The survey was conducted at the University Hospital in Erlangen (Germany) during the period from December 2021 to May 2022. Participants were provided with full information on the study, they gave informed consent to participate and data was pseudonymized. A total of 227 women received a questionnaire.

### Questionnaire design

The questionnaire was designed in German language (Supplementary Material S1) and consisted of four parts: (a) participants’ characteristics: this covered questions on demographics, gravidity, parity, and previous experience with the use of electronic devices (16 questions); (b) wearables: this part contained questions designed to assess participants’ attitudes toward these devices and the extent of their openness to using them during their pregnancy (27 questions), including the following previously published questionnaire constructs: Attitude (Toward Using Technology),^24–26^ Behavioral Intention (to Use),^27,28^ Performance (Perceived) Expectancy,^27,29^ Effort Expectancy,^27,30^ Social Influence,^27,31^ Health Consciousness^32,33^ and Perceived Privacy Risk^34^; (c) Technology Readiness Index 2.0 (TRI 2.0; 10 questions); and (d) German Version of the PRAQ-R2 (10 questions).^35^

One of the most frequently used measures of PrA worldwide is the Pregnancy-Related Anxiety Questionnaire (PRAQ)^36^ and adaptions thereof: Huizink et al. revised the PRAQ, creating a feasible abbreviated 10-item version that did not distinguish respondents by parity (PRAQ-R2)^37^ and which Mudra et al. subsequently translated into German.^35^ The questionnaire covers three subscales for the assessment of birth- or child-specific worries, alongside potential concerns the woman may have in relation to herself. These subscales are: (a) “fear of giving birth” (FoGB); (b) “worries about bearing a physically or mentally handicapped child” (WaHC); (c) “concerns about own appearance” (CoA).

The questions on wearables, the TRI 2.0, and the PRAQ-R2 were answered on a five-point Likert scale ranging from “strongly disagree” (1) to “strongly agree” (5), or, analogously, from “never” (1) to “very often” (5). The results of the TRI 2.0 questionnaire are not part of this publication, which focusses solely on the anxiety sub-project. Patients were excluded from analysis if they had responded to less than 70% (30 of 43) of the questions on participants’ characteristics and wearables. The PRAQ-R2 questionnaire was only included if all items were completed.

### Data analysis

We conducted a descriptive statistical analysis of the participants’ characteristics and their responses to the questionnaire using Microsoft Excel LTSC MSO (version 16.0.14332.20810) and IBM SPSS (version 29.0.1.0). The PRAQ-R2 consists of 10 items in total, with a maximum score of 50 points across all items. It addresses three main dimensions of pregnancy-related anxiety: “fear of giving birth” (FoGB, three items), “worries about bearing a handicapped child” (WaHC, four items), and “concerns about own appearance” (CoA, three items). The cut-off was defined according to Sanni et. al.^38^ Participants equal or above the 75^th^ percentile of the total scores in each group were defined as the “high-anxiety” group (cut-off values: FoGB ≥ 11 points, WaHC ≥ 15 points, CoA ≥ 11 points, PrA ≥ 37.5 points) and below as the “low-anxiety” control group.

Analysis of associations between general characteristics and anxiety groups were done using Pearson's Chi-squared tests for categorical values and Mann–Whitney *U* tests for continuous values to an exact significance level of *P* = .05. Associations between the anxiety groups and the attitude, intention and willingness to use smart devices were analyzed using Mann–Whitney *U* tests to an exact significance level of *P* = .05. The Mann–Whitney *U* test was chosen, as the data did not meet the assumptions of normality (Likert scale data). Given the exploratory nature of the study, no correction of multiple testing was applied to avoid overlooking potentially relevant associations. Therefore, reported *P*-values should be interpreted with caution.

## Results

Of the 227 questionnaires distributed, 210 (92.5%) were analyzed for participants’ characteristics, questions on wearables and pregnancy-related anxiety (Supplementary Material S2). Eight questionnaires (3.5%) were not returned, four were categorized as incomplete (1.8%; fewer than 70% of the items in the general section completed), four did not complete the PRAQ-R2 (1.8%) and one woman (0.4%) was retrospectively excluded due to irregular study inclusion. Based on the questionnaire, 191 (91%) women were categorized as having low PrA, while 19 (9%) were categorized as having high PrA.

### General characteristics of participants

Table 1 provides an overview of participant characteristics. The youngest participant enrolled in the study was 22 years old, the oldest 43 years, and the median age was 32 years, and 86.6% of the respondents had an educational status equal or above a high school diploma. One hundred and fifteen (55.0%) were, at the time of responding, not working predominantly because they either begun their maternity leave or were subject to a medical prohibition on working due to their pregnancy; 94 (45.0%) were working full- or part-time. One hundred and fifty-one of the participants were married (72.6%). The pregnancy was the first for 84 (40.0%) participants and 152 (72.4%) women were in their 25^th^ week of pregnancy or later; 55 (26.2%) had previously suffered at least one miscarriage. Most of the participants (81.9%) had become pregnant without fertility treatment (Table 1). Asking women about their previous app usage revealed that most of them did not use fitness apps (76.0%) and have not used a fitness tracker before (53.6%), but most of them used pregnancy apps (68.1%). Statistical analysis showed no significant differences between low and high PrA (*P* > .05) (Table 1).

**Table 1.** General characteristics of 210 participants.

Characteristic	Total (*n* = 210)	Low PrA (*n* = 191)	High PrA (*n* = 19)	*P*-Value^a^
Median age in years (Min, Max)	32 (22, 43)	31 (22, 42)	32 (22, 43)	.17
Educational status, *n* (%)				.75
<High school diploma	28 (13.40)	25 (13.09)	3 (15.79)	
≥High school diploma	181 (86.60)	165 (86.39)	16 (84.21)	
Missing	1	1	0	
Employment status, *n* (%)				.10
Not working	115 (55.02)	108 (56.54)	7 (36.84)	
Working	94 (44.98)	82 (42.93)	12 (63.16)	
Missing	1	1	0	
Marital status, *n* (%)				.13
Single	57 (27.40)	49 (25.93)	8 (42.11)	
Married	151 (72.60)	140 (74.07)	11 (57.89)	
Missing	2	2	0	
Gestational week, *n* (%)				.22
1–24	58 (27.62)	55 (28.80)	3 (15.79)	
25–40+	152 (72.38)	136 (71.20)	16 (84.21)	
Gravidity, *n* (%)				.84
1	84 (40.00)	76 (39.79)	8 (42.11)	
>1	126 (60.00)	115 (60.21)	11 (57.89)	
Parity, *n* (%)				.12
Nulliparous	97 (46.19)	85 (44.50)	12 (63.16)	
Multiparous	113 (53.81)	106 (55.50)	7 (36.84)	
Previous miscarriages, *n* (%)				.58
None	155 (73.81)	142 (74.35)	13 (68.42)	
≥1	55 (26.19)	49 (25.65)	6 (31.58)	
Fertility treatment, *n* (%)				.78
No	172 (81.90)	156 (81.68)	16 (84.21)	
Yes	38 (18.10)	35 (18.32)	3 (15.79)	
Use of fitness apps				.33
No	158 (75.96)	146 (76.84)	12 (66.67)	
Yes	50 (24.04)	44 (23.16)	6 (33.33)	
Missing	2	1	1	
Use of pregnancy apps				.90
No	67 (31.90)	61 (31.94)	6 (33.33)	
Yes	142 (68.10)	130 (68.06)	12 (66.67)	
Missing	1	0	1	
Previous use of fitness tracker				.82
No	110 (52.63)	101 (52.88)	9 (50.00)	
Yes	99 (47.37)	90 (47.12)	9 (50.00)	
Missing	1	0	1	

a Pearson's Chi-squared test for categorical values; Mann–Whitney *U* test for continuous values.

Values are expressed as numbers and percentages with respect to total sample size.

### Pregnancy-related anxiety: PRAQ-R2

**Figure 1.**
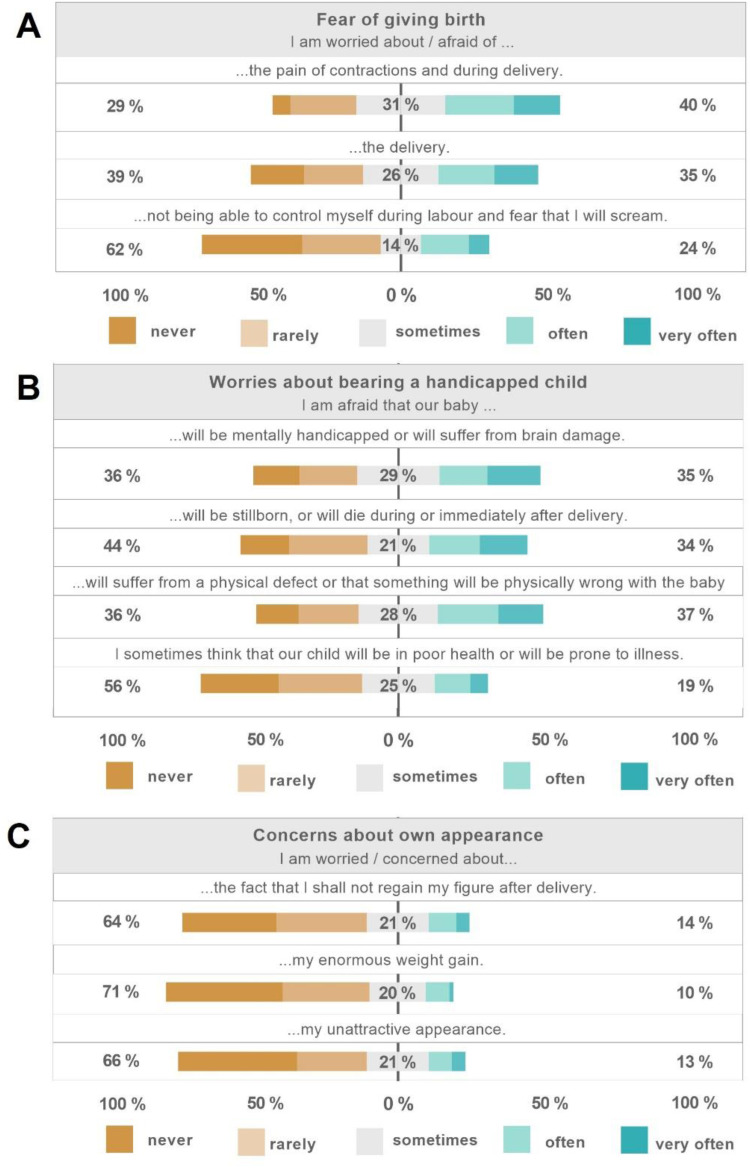
Evaluation of the pregnancy-related anxiety questionnaire (PRAQ-R2). (A) Fear of giving birth, (B) Worries about bearing a handicapped child and (C) Concerns about own appearance. The reported percentages represent aggregated responses, where “never” and “rarely” are combined, as well as “often” and “very often”.

Figure 1 presents the respective responses from participants to the PRAQ-R2. Of the participants who completed the PRAQ-R2, 69% (*n* = 144) were classified as having a “low FoGB” and 31% (*n* = 44) as “high FoGB” based on the sum of the respective subcategories (Supplemental Material S3, Figure 1(A)). For WaHC, 62% (*n* = 152) scored in the low and 38% (*n* = 58) in the high category (Supplemental Material S3, Figure 1(B)). Regarding CoA, 89% (*n* = 187) of women were categorized as low and 11% (*n* = 23) as high (Supplemental Material S3, Figure 1(C)). When considering all domains collectively, 91% (*n* = 191) of respondents were categorized as having a low PrA level and 9% (*n* = 19) as high (Supplementary Material S3).

### Associations between pregnancy-related anxiety among participants and their willingness to use a smart device in the context of their prenatal care

We aimed to analyze differences between high- and low-anxiety groups and the participants’ anticipated frequency of use, attitude toward smart devices, and behavioral intention to use smart devices as a part of their prenatal care (Table 2).

**Table 2.** Pregnancy-related anxiety groups and associations with participants’ willingness to use a smart device in the context of their prenatal care.

Variable	High PrA (*n* = 19), median (IQR)	Low PrA (*n* = 191), median (IQR)	*P*-Value	Missing
Frequency of use^a^: “How often would you be willing to use this type of device to better monitor your health during pregnancy and your unborn child?”				
Smartwatch	5.00 (3.00–5.00)	4.00 (2.00–5.00)	.03	1
Contraction Counter	4.00 (3.00–5.00)	3.00 (2.00–4.00)	.008	6
BPM Device	3.00 (2.00–3.00)	3.00 (2.00–4.00)	.11	7
Sleep Tracker	3.00 (2.00–3.00)	3.00 (2.00–3.00)	.09	10
Attitude^b^: “I think that using this type of device in prenatal care is a good idea. “				
Smartwatch	4.00 (4.00–5.00)	4.00 (3.00–5.00)	.07	1
Contraction Counter	4.00 (4.00–5.00)	4.00 (3.00–4.00)	.002	1
BPM Device	5.00 (4.00–5.00)	4.00 (3.00–4.25)	.003	1
Sleep Tracker	5.00 (3.00–5.00)	3.00 (3.00–4.00)	.002	1
Intention to use^b^: “Will you use this type of device for prenatal care if you were given the opportunity?”				
Smartwatch	4.00 (4.00–5.00)	4.00 (3.00–4.00)	.02	
Contraction Counter	4.00 (3.00–5.00)	3.00 (3.00–4.00)	.02	
BPM Device	5.00 (3.00–5.00)	4.00 (3.00–4.00)	.003	
Sleep Tracker	4.00 (3.00–5.00)	3.00 (2.00–4.00)	.007	

IQR: interquartile range; *n*: number; PrA: Pregnancy-related anxiety.

Analysis of associations was done using Mann–Whitney *U* tests to an exact significance level of *P* = .05. Values are expressed as median (interquartile range) or numbers.

a Likert scale options: 1 = fewer, 2 = 1–4 times per week, 3 = 1 time per day, 4 = multiple times per day, 5 = as often as possible; option 4 and 5 not available for Sleep Tracker.

b Likert scale options: 1 = strongly disagree, 2 = disagree, 3 = neutral, 4 = agree, 5 = strongly agree.

Women with high PrA levels tend to use a smartwatch (median score 5.00; interquartile range (IQR) 3.00–5.00 vs. 4.00; IQR 2.00–5.00, *P* = .03) and a smart contraction counter (median score 4.00; IQR 3.00–5.00 vs. 3.00; IQR 2.00–4.00, *P* = .008) more often than women with a low level of PrA (Table 2). Moreover, the high PrA group stated a more positive attitude towards using a smart contraction counter (median score 4.00; IQR 4.00–5.00 vs. 4.00; IQR 3.00–4.00, *P* = .002), a smart BPM device (median score 5.00; IQR 4.00–5.00 vs. 4.00; IQR 3.00–4.25, *P* = .003), and a smart sleep tracker (median score 5.00; IQR 3.00–5.00 vs. 3.00; IQR 3.00–4.00, *P* = .002). When asked, if they would use the smart devices in their prenatal care routine if they were given the opportunity, a higher intention to employ these smart devices was demonstrated by women with high PrA levels (*P* < .02) (Table 2).

Women who reported worries about bearing a handicapped child showed more frequent willingness to use a smart device like a smartwatch (median score 5.00; IQR 3.00–5.00 vs. 4.00; IQR 2.00–5.00; *P* = .02), contraction counter (median score 4.00; IQR 3.00–5.00 vs. 3.00; IQR 2.00–4.00; *P* < 0.001), BPM device (median score 3.00; IQR 2.00–4.00 vs. 3.00; IQR 2.00–4.00; *P* = .04) or a sleep tracker (median score 3.00; IQR 2.00–3.00 vs. 2.00; IQR 1.00–3.00; *P* = .03) to monitor their and their unborn child's health during pregnancy (Supplementary Material 4). Women who fear giving birth stated that they would use a contraction counter more often (median score 3.00; IQR 2.00–5.00 vs. 3.00; IQR 2.00–4.00; *P* = .007). Furthermore, participants of the “high FoGB” group had a higher tendency to value using a smartwatch (median score 4.00; IQR 3.00–5.00 vs. 4.00; IQR 3.00–5.00; *P* = .04), contraction counter (median score 4.00; IQR 4.00–5.00 vs. 4.00; IQR 3.00–4.00; *P* < 0.001) or a sleep tracker (median score 4.00; IQR 3.00–5.00 vs. 3.00; IQR 3.00–4.00; *P* = .005) as a good idea compared to those of the “low FoGB” group (Supplemental Material S4).

Due to the capacity of smartwatches to measure a broad variety of health parameters of significance (e.g. sleep quality and duration, physical activity, electrocardiogram (ECG) measurements, and heart rate), participants’ perceptions of a smartwatch's usefulness in the context of prenatal care were assessed in detail (Table 3).

**Table 3.** Pregnancy-related anxiety group and associations with the perceived usefulness (performance expectancy) of a smartwatch's use in the context of prenatal care.

Variable	High PrA (*n* = 19), median (IQR)	Low PrA (*n* = 191), median (IQR)	*P*-Value
I think it could be useful.	4.00 (4.00–5.00)	4.00 (3.00–5.00)	.04
It could help to accomplish health-related aims in prenatal care more quickly.	5.00 (3.00–5.00)	4.00 (3.00–4.00)	.01
It could help me with my daily health checks during pregnancy.	5.00 (3.00–5.00)	4.00 (3.00–4.00)	.02
Performance expectancy	14.00 (10.00–15.00)	12.00 (10.00–13.00)	.02

IQR: interquartile range; *n*: number; PrA: Pregnancy-related anxiety. Likert scale options: 1 = strongly disagree, 2 = disagree, 3 = neutral, 4 = agree, 5 = strongly agree.

Questions were answered on a five-point Likert scale. Analysis of associations was done using Mann–Whitney *U* tests to an exact significance level of *P* = .05. Values are expressed as median (interquartile range) or numbers.

Women with a high level of PrA showed a significantly higher tendency to consider a smartwatch generally useful for prenatal care (median 4.00; IQR 4.00–5.00 vs. 4.00; IQR 3.00–5.00, *P* = .04), helpful for accomplishing health-related aims more quickly (median 5.00; IQR 3.00–5.00 vs. 4.00; IQR 3.00–4.00, *P* = .01) and for the assistance with daily health checks (median 5.00 (IQR 3.00–5.00) vs. 4.00; IQR 3.00–4.00, *P* = .02) compared to women of the “Low PrA” group (Table 3). The overall performance expectancy was significantly higher for women with high PrA (median 14.00; IQR 10.00–15.00 vs. 12.00; IQR 10.00–13.00, *P* = .02). Women categorized within the “High FoGB” group showed a significantly higher performance expectancy than women with low FoGB (median 12.00; IQR 10.00–15.00 vs. 12.00; IQR 9.25–13.00, *P* = .02) (Supplementary Material S5). No significant differences were observed between high and low WaHC, and high and low CoA (Supplementary Material S5).

## Discussion

This study represents, to our knowledge, the first analysis of the association between pregnancy-related anxiety among women from week 9 of pregnancy onward and their willingness to use wearables and smart devices such as smartwatches, smart contraction counters, smart BPM devices, and sleep trackers to supplement their face-to-face prenatal care appointments. We found that the majority of respondents had a positive attitude toward the use of smart devices, especially smartwatches and contraction counters, to supplement their prenatal care, and could envisage themselves using them. Our findings are in line with other studies reporting positive views of wearable ECG technologies for monitoring of maternal and fetal health,^18^ a willingness to wear devices as often as possible,^18^ and openness toward the use of smart devices for self-monitoring during pregnancy.^17^ A survey revealed that a large majority of pregnant women (75%, *n* = 78) expressed willingness to modify their behaviors during pregnancy if they received personalized recommendations through their smartphones, based on factors like height, weight, and physiological monitoring. Additionally, when considering different scenarios for wearing sensors to monitor health and environmental factors, pregnancy health monitoring was the top choice, with 76% (*n* = 79).^17^Further, the SMART Start study demonstrated that pregnant women are open to participating in prenatal testing at home, and a consistently high level of engagement with smart devices over several weeks highlights their practicality and feasibility for routine prenatal care.^16^ This said, some skepticism around the devices remains among pregnant women.^39,40^ Clinicians may be able to counter these concerns by recommending the use of smart devices and reviewing the health data they generate.^39,40^ It was also found that there is an overall positive attitude towards mHealth technologies and wearables for monitoring pregnant women's health and well-being among clinical healthcare professionals.^40^ In this study, it is interesting that the women did not differ on high versus low pregnancy-related anxiety dependent on parity, prior miscarriage or fertility treatment as it is often seen in other studies.^41–43^ This might be attributed to several factors: It is possible that other variables, such as maternal age, socioeconomic status, or educational level played a more influential role in this particular sample, masking the effects typically observed with parity or reproductive history.^41,44^ Another consideration is that recruitment and assessment timing may have reduced the usual impact of reproductive history. For example, anxiety related to previous miscarriages may diminish after a certain gestational threshold or as women receive more psychosocial or medical support since more than two thirds of the women were between 25 and 40 weeks pregnant at the time of the survey.^41,44^

A concept analysis of pregnancy anxiety identified two main components: general fear of childbirth and specific concerns. The most frequently reported pregnancy-related anxiety items were pain and concerns about not having a “normal” delivery, as well as fetal health (damage to the fetus, fetal abnormality, and fetal health and wellbeing in general).^45^ Our findings echo these considerations; 31% (66 of 210) of our respondents were categorized, as having high levels of fear of giving birth, while 28% (58 of 210) had high levels of worries about bearing a handicapped child. The mean PRAQ-R2 score in this study was 26.11 ± 8.14 (median score 26.00, IQR 21.00–31.00), which is in good agreement with other findings.^46–48^ Notably, we observed that these women reported willingness to use smart devices in their prenatal care significantly more often than non-anxious women, and also showed significantly higher overall willingness in this context. This might appear in line with non-pregnancy-specific findings indicating a significant effect of wearables in terms of reducing distress and improving mental health.^49,50^ Other studies report contrasting results; pregnant participants in a qualitative interview study stated their concern that constant monitoring might represent an additional source of anxiety where measurements or values were out with optimum ranges.^40^ Further, for individuals with pathological anxiety—marked by frequent, uncontrollable, and excessive worry—these devices might reinforce reassurance-seeking behavior, potentially maintaining or worsening their symptoms rather than relieving them.^18^ We surmise from this that constant monitoring may be of benefit to women with high levels of anxiety, potentially helping to ameliorate their concerns and reduce stress, but for pregnant women with clinically elevated anxiety or anxiety disorders it could also contribute to the maintenance or even worsening of symptoms. Similarly, it may tend toward having a distressing effect on low-anxiety women. It is recommended that future studies encompass a more precise evaluation of the anxiety levels experienced by pregnant women. This will facilitate the identification of those who would derive the greatest benefit from continuous monitoring via wearable devices, as opposed to those who would be more susceptible to risk as a consequence. We further believe that our findings underline the importance of healthcare professionals’ engagement with women around the use of wearables. In view of our finding that pregnant women value a professional opinion in this context, the issuance of recommendations may lead to greater openness toward wearables and more confidence in the health data they generate, with the result of a reduction in pregnancy anxiety and a greater sense of security among pregnant women. A significant aspect of wearables’ use in pregnancy relates to the manifold changes a woman's body undergoes during this period, including changes in vital parameters. Wearables currently widely available are not trained to take physiological changes during pregnancy into account, making it difficult to interpret data entirely accurately^1,4^ and thus carrying the risk that the data they generate may cause a woman to worry unnecessarily about the state of her health or her baby's. The sharing and discussion of data from wearables with healthcare professionals during the course of routine face-to-face appointments may avoid situations in which women misinterpret values. To tackle these challenges, best practices may include recommending wearables only when there is a clear health goal or metric to monitor, and ensuring that patients are educated about both the limitations and utility of specific devices. Providers should discuss which devices are clinically validated for pregnancy and be transparent about potential inaccuracies, to minimize unnecessary anxiety. Research is still needed to establish guidelines that reduce iatrogenic effects—for example, misleading reassurance or increased anxiety stemming from unclear data—by studying device accuracy, patient education, and management strategies for abnormal readings. Further, local standards of data quality, promoting interoperable systems, ensuring equitable access, and enhancing the representativity of datasets are recommended as key steps to mitigate these pitfalls.^19^

Our study, however, has some limitations. First, the study solely consisted of a questionnaire. Women actually using such smart devices might react differently after the use and their perception might change. Second, participants who chose to respond to the questionnaire may have been more interested in technology and digital health solutions, potentially leading to an overestimation of willingness to use smart devices during pregnancy. Third, one of the items assessing the willingness included the wording “to better monitor your health during pregnancy and your unborn child”. The use of the term “better” may have introduced bias, as it implicitly frames the device as an improvement, potentially elevating perceived usefulness and, consequently, willingness to use it. Forth, the sample population may not be representative of all pregnant women, as the study was conducted at the University Hospital Erlangen, we report a general higher frequency of patients with a top-level educational background compared to other cities and factors such as educational level and socioeconomic background could influence perceptions of smart device use.

Future research should involve actual use of smart devices by pregnant women, collecting both quantitative and qualitative data over time to capture real-world experiences and changes in perceptions post-use. Further, to overcome selection bias multi-center studies including general practitioners and gynecologists in private practice beyond single-center studies could help to recruit a more representative, diverse sample across different socioeconomic backgrounds, educational levels, and geographic areas. Overall, research should focus on integrating data with prenatal care systems to complement – not to replace–traditional visits, improving clinical decision-making and personalized care.

## Conclusion

In summary, our study aimed to ascertain any links between pregnancy-related anxiety and pregnant women's willingness to use smart devices as a supplement to their prenatal care. Overall, our data suggest that pregnant women are largely open-minded toward the use of various smart devices in the context of prenatal care. Notwithstanding the potential benefits of smart devices for pregnancy monitoring, further research in this area is required. Our study may represent an initial step toward gaining a full picture of attitudes of anxious pregnant women toward these devices and their willingness to use them; acceptability and feasibility studies could usefully follow, alongside the pursuit of technological advancements in pregnancy-specific wearables and studies around their impact on pregnancy care.

## Supplemental Material

sj-pdf-1-dhj-10.1177_20552076251406652 - Supplemental material for Using smart devices for prenatal care: Assessing the willingness among women with pregnancy-related anxiety Supplemental material, sj-pdf-1-dhj-10.1177_20552076251406652 for Using smart devices for prenatal care: Assessing the willingness among women with pregnancy-related anxiety by Stefanie Altmannshofer, Filip Weidenthaler, Adriana Titzmann, Constanza A Pontones, Nina Danzberger, Katharina M Jaeger, Michael Nissen, Heike Leutheuser, Bjoern M Eskofier, Peter A Fasching, Matthias W Beckmann and Hanna Huebner in DIGITAL HEALTH

sj-docx-2-dhj-10.1177_20552076251406652 - Supplemental material for Using smart devices for prenatal care: Assessing the willingness among women with pregnancy-related anxiety Supplemental material, sj-docx-2-dhj-10.1177_20552076251406652 for Using smart devices for prenatal care: Assessing the willingness among women with pregnancy-related anxiety by Stefanie Altmannshofer, Filip Weidenthaler, Adriana Titzmann, Constanza A Pontones, Nina Danzberger, Katharina M Jaeger, Michael Nissen, Heike Leutheuser, Bjoern M Eskofier, Peter A Fasching, Matthias W Beckmann and Hanna Huebner in DIGITAL HEALTH

sj-docx-3-dhj-10.1177_20552076251406652 - Supplemental material for Using smart devices for prenatal care: Assessing the willingness among women with pregnancy-related anxiety Supplemental material, sj-docx-3-dhj-10.1177_20552076251406652 for Using smart devices for prenatal care: Assessing the willingness among women with pregnancy-related anxiety by Stefanie Altmannshofer, Filip Weidenthaler, Adriana Titzmann, Constanza A Pontones, Nina Danzberger, Katharina M Jaeger, Michael Nissen, Heike Leutheuser, Bjoern M Eskofier, Peter A Fasching, Matthias W Beckmann and Hanna Huebner in DIGITAL HEALTH

sj-docx-4-dhj-10.1177_20552076251406652 - Supplemental material for Using smart devices for prenatal care: Assessing the willingness among women with pregnancy-related anxiety Supplemental material, sj-docx-4-dhj-10.1177_20552076251406652 for Using smart devices for prenatal care: Assessing the willingness among women with pregnancy-related anxiety by Stefanie Altmannshofer, Filip Weidenthaler, Adriana Titzmann, Constanza A Pontones, Nina Danzberger, Katharina M Jaeger, Michael Nissen, Heike Leutheuser, Bjoern M Eskofier, Peter A Fasching, Matthias W Beckmann and Hanna Huebner in DIGITAL HEALTH

sj-docx-5-dhj-10.1177_20552076251406652 - Supplemental material for Using smart devices for prenatal care: Assessing the willingness among women with pregnancy-related anxiety Supplemental material, sj-docx-5-dhj-10.1177_20552076251406652 for Using smart devices for prenatal care: Assessing the willingness among women with pregnancy-related anxiety by Stefanie Altmannshofer, Filip Weidenthaler, Adriana Titzmann, Constanza A Pontones, Nina Danzberger, Katharina M Jaeger, Michael Nissen, Heike Leutheuser, Bjoern M Eskofier, Peter A Fasching, Matthias W Beckmann and Hanna Huebner in DIGITAL HEALTH

## Abbreviations

BPM: blood pressure monitor
CoA: concerns about own appearance
ECG: electrocardiogram
FoGB: fear of giving birth
PrA: pregnancy-related anxiety
PRAQ: pregnancy-related anxiety questionnaire
PRAQ-R2: pregnancy-related anxiety questionnaire—revised 2
TRI 2.0: technology readiness index 2.0
WaHC: worries about bearing a (mentally or physically) handicapped child
