# Supplementary material for: Using smart devices for prenatal care: Assessing the willingness among women with pregnancy-related anxiety
Source: Digit Health. 2026 Jan 27;12:20552076251406652. doi: 10.1177/20552076251406652 (PMC12847662; doi:10.1177/20552076251406652)
Supplement: sj-pdf-1-dhj-10.1177_20552076251406652 - Supplemental material for Using smart devices for prenatal care: Assessing the willingness among women with pregnancy-related anxiety [file sj-pdf-1-dhj-10.1177_20552076251406652.pdf]

UMFRAGE ZUM THEMA DIGITALISIERUNG IN DER SCHWANGERSCHAFTSVORSORGE

Evaluation der Bereitschaft zur Nutzung komplexer sensorischer Anwendungen in der Schwangerschaftsvorsorge

Datum:
Patienten-ID:

Vielen Dank, dass Sie unseren Fragebogen zum Thema Digitalisierung in der Schwangerschaftsvorsorge ausfüllen möchten.

Viele Faktoren beeinflussen die Bereitschaft der Schwangeren neue elektronische Messgeräte in der Schwangerschaftsvorsorge zu nutzen. In dieser Umfrage geht es vor allem darum, verschiedene Einflussfaktoren auf diese Bereitschaft zu untersuchen.

Diese Umfrage gehört zu der Studie SMART Start.

SMART Start ist ein interdisziplinäres Forschungsprojekt von Medizin, Informatik, Ethik, Psychologie und Gesundheitsökonomie des Universitätsklinikums Erlangen (UKER) und der Friedrich-Alexander-Universität Erlangen-Nürnberg (FAU). Das Projekt ist gefördert durch das Bundesministerium für Gesundheit.

Diese Umfrage beansprucht ca. 15 Minuten Ihrer Zeit.

Die erhobenen Angaben dienen ausschließlich als Basis für eine wissenschaftliche Arbeit.
Ihre Daten werden vertraulich behandelt.

Bitte beantworten Sie alle Fragen bis zum Ende der Umfrage, auch wenn Sie in näherer Zukunft nicht beabsichtigen die neuen elektronischen Messgeräte in der Schwangerschaft zu verwenden.

Bei Fragen können Sie sich gerne an das Studienteam wenden.

Herzlichen Dank für Ihre Unterstützung!

Gefördert durch:

Bundesministerium
für Gesundheit

aufgrund eines Beschlusses
des Deutschen Bundestages

1. Demografische Fragen

1.1. **Wie alt sind Sie?**

____

1.2. **Was ist Ihre höchste absolvierte Ausbildung?**

☐ kein Abschluss
☐ Grund-/Hauptschulabschluss
☐ mittlere Reife
☐ Abitur
☐ abgeschlossene Ausbildung
☐ Fachhochschulabschluss
☐ Hochschulabschluss
☐ Promotion
☐ Habilitation
☐ andere

1.3. **Welche der folgenden Kategorien beschreibt am besten Ihren aktuellen Beschäftigungsstatus?**

☐ Vollzeit (> 35h pro Woche)
☐ Teilzeit
☐ Schülerin/Studentin
☐ arbeitsunfähig
☐ Hausfrau
☐ arbeitslos
☐ arbeitssuchend
☐ Mutterschutz

1.4. **Sind Sie derzeit verheiratet, verwitwet, geschieden, getrennt oder ledig?**

☐ verheiratet
☐ verwitwet
☐ geschieden
☐ getrennt
☐ ledig
☐ keine Angabe

1.5. **Wie lange ist Ihre Anfahrtszeit in die Klinik?** (bitte in Minuten angeben)

____

2. Fragen zur Schwangerschaft

2.1. **In welcher Schwangerschaftswoche befinden Sie sich derzeit?**

☐ 1.-12.
☐ 13.-24.
☐ 25.-40.
☐ über 40.

2.2. **Um die wievielte Schwangerschaft** (inklusive Fehlgeburten, Schwangerschaften außerhalb der Gebärmutter und Schwangerschaftsabbrüchen) **handelt es sich bei Ihnen aktuell?**

☐ 1.
☐ 2.
☐ 3.
☐ 4.
☐ 5.
☐ 6. und mehr

2.3. **Wie viele Kinder haben Sie geboren?** (inklusive Totgeburten, Geburt von Zwillingen ist als eine Geburt zu verstehen)

☐ Keines
☐ 1
☐ 2
☐ 3
☐ 4
☐ 5 und mehr

2.4. **Wie viele Fehlgeburten hatten Sie?**

☐ Keine
☐ 1
☐ 2
☐ 3
☐ 4
☐ 5 und mehr

2.5. **Wurden Sie jemals wegen Kinderwunsch behandelt?**

☐ Ja
☐ Nein

Falls ja, mit folgender Methode(n): ____________________________

3. Fragen zur Technologieerfahrung

3.1. **Besitzen Sie ein Smartphone oder ein Tablet?**

☐ Ich besitze Beides
☐ Ich besitze nur ein Smartphone
☐ Ich besitze nur ein Tablet
☐ Nein, ich besitze weder ein Smartphone noch ein Tablet

3.2. **Haben Sie zu Hause WLAN oder unbegrenztes Datenvolumen auf Ihrem Smartphone?**

☐ Ich habe Beides
☐ Ich habe nur unbegrenztes Datenvolumen
☐ Ich habe nur WLAN
☐ Nein, nichts davon

3.3. **Nutzen Sie Fitness-Apps?** (wie z.B. Sworkit, Nike+, Runtastic, Strava, iYoga+, 7Mind)

☐ Ja
☐ Nein

3.4. **Nutzen Sie Schwangerschafts-Apps?** (wie z.B. Clue, WomanLog, Keleya, Embryotox, Schwangerschaft+)

☐ Ja
☐ Nein

Falls ja, folgende Apps: ______________________________

3.5. **Besitzen Sie einen Fitness-Tracker (Gesundheits-Armband) oder eine Smartwatch?**

☐ Ja, ich verwende derzeit eine Smartwatch oder einen Fitness-Tracker
☐ Ja, ich benutze sie aber nicht mehr
☐ Nein, ich habe noch nie eine Smartwatch oder einen Fitness-Tracker benutzt

3.6. **Falls Sie einen Fitness-Tacker/Smartwatch besitzen, welche Funktionen verwenden Sie oder haben Sie verwendet?** (Mehrfachantworten sind möglich)

☐ Sportaktivitäten
☐ Schlafüberwachung
☐ Kalorienverbrauchsmessung
☐ Andere: _________________________
☐ Herzfrequenzmessung
☐ EKG-Messung
☐ Blutdruckmessung

4. Fragen zu Wearables

Das vorrangige Ziel der Schwangerschaftsvorsorge ist die frühzeitige Erkennung und Betreuung von Risikoschwangerschaften.

In der frauenärztlichen Praxis werden regelmäßig Vitalzeichen wie Blutdruck, Herzfrequenz, Temperatur und körperliche Parameter wie Gewicht erfasst. Es werden auch einfache Untersuchungen des Urins durchgeführt. Ultraschalluntersuchungen und in den späteren Schwangerschaftswochen regelmäßig durchgeführte Aufzeichnungen der kindlichen Herzfrequenz, bzw. der Wehentätigkeit sind ebenfalls ein fester Bestandteil der Schwangerschaftsvorsorge.

Es wäre jedoch möglich, einen Großteil dieser Untersuchungen einfach und bequem von Zuhause durchzuführen.
Die Übertragung der gemessenen Werte könnte automatisch über eine App auf Ihrem Handy an Ihren behandelnden Arzt erfolgen.

Es gibt aber auch weitere Parameter, die einen wichtigen Einfluss auf den Schwangerschaftsverlauf haben, deren Verfolgung aber noch kein fester Bestandteil der Schwangerschaftsvorsorge ist.
Schnarchen und nächtliches Erwachen, um nach Luft zu schnappen nimmt im Laufe der Schwangerschaft zu. Es ist belegt, dass Schlafstörungen in der Schwangerschaft mit erhöhtem Frühgeburtsrisiko oder geringem Geburtsgewicht zusammenhängen. Eine häusliche Überwachung Ihres Schlafes ist bereits heutzutage möglich.

Im Folgenden stellen wir Ihnen vier Geräte vor.

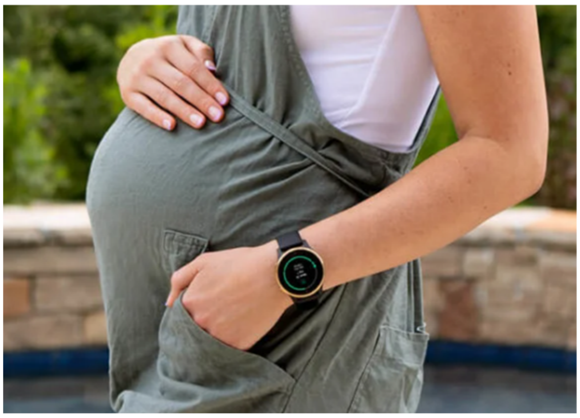	**SMARTWATCH**<br><br>Eine Smartwatch ist eine elektronische Armbanduhr, die über zusätzliche Sensoren verfügt und die gemessenen Daten über Bluetooth an kompatible Smartphones überträgt. Eine Smartwatch bietet viele Funktionen, wie z.B.<br>• EKG-Funktion<br>• Verfolgen von Sportaktivitäten<br>• Monitoring des Schlafverhaltens<br>• Messung des Kalorienverbrauches<br>• Überwachung der Herzfrequenz<br>• Messung der Sauerstoffsättigung<br>• Erinnerungsfunktion

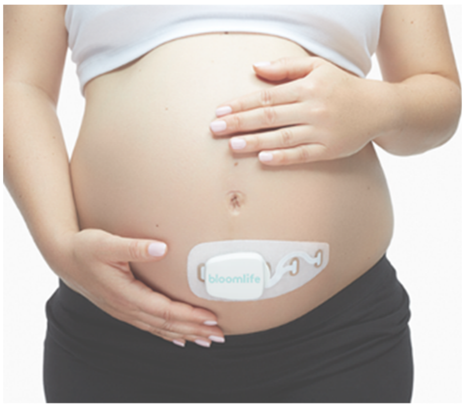	Smartphone-koppelbarer **KONTRAKTIONSZÄHLER**<br><br>Ein Kontraktionszähler ermöglicht, Wehentätigkeit der Gebärmutter genau zu messen und aufzuzeichnen. Bereits heutzutage gibt es kabellose und mit Smartphone koppelbare Kontraktionszähler, die auch zu Hause verwendet werden können.<br><br>Zukünftig werden solche Geräte auch über CTG-Funktion (Messung der kindlichen Herztöne) verfügen und damit genau vorhersagen zu können, wie es Ihrem ungeborenen Kind geht.

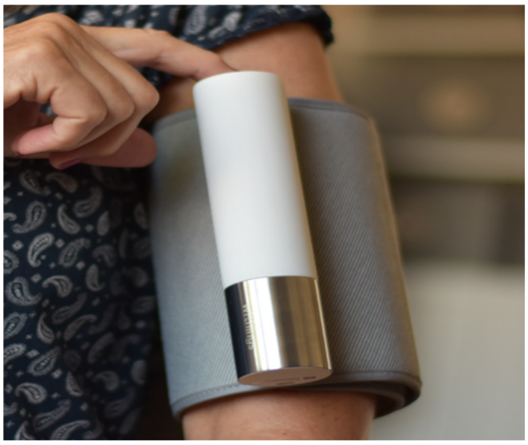	Smartphone-koppelbares **BLUTDRUCKMESSGERÄT**<br><br>Automatische, Smartphone-koppelbare Blutdruckmessgeräte ermöglichen einfache Heimblutdruckmessungen.<br><br>Mit dem Gerät ist auch Herzfrequenz- und EKG-Messung möglich.<br><br>Außer Bluthochdruck können damit auch Herzrhythmusstörungen festgestellt werden.

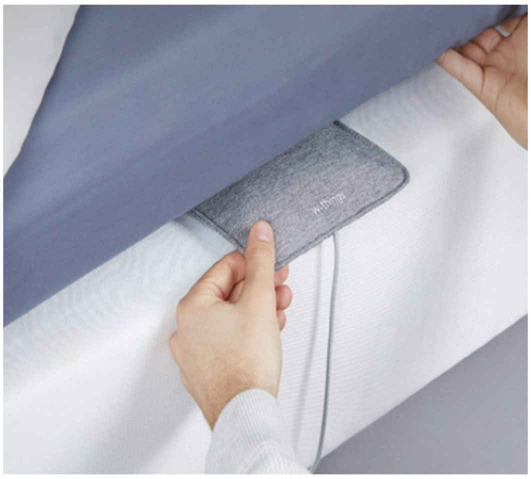	**SCHLAFSENSOR**<br><br>Bei diesem Schlafsensor handelt es sich um eine etwa 60 x 20 cm große und 2 cm dicke Matte, die unter die Matratze gelegt wird.<br><br>Die eingebauten Sensoren ermöglichen eine einfache Überwachung der Schlafdauer und Schlafqualität.

Jetzt stellen Sie sich vor, Sie würden in der Schwangerschaft die oben genannten Geräte zu Hause nutzen und die gemessenen Daten an Ihren Frauenarzt oder Ihre Frauenärztin übermitteln.

4.1. **Wie oft wären Sie bereit, folgende Geräte zu verwenden, um Ihre Gesundheit in der Schwangerschaft und Ihr ungeborenes Kind besser überwachen zu lassen?**

	**So oft wie möglich**	**Mehrmals am Tag**	**1x täglich**	**1-4x in der Woche**	**Weniger oft**
**Smartwatch** (mit EKG-Funktion)	☐	☐	☐	☐	☐
**Kontraktionszähler** (zum Aufkleben auf den Bauch)	☐	☐	☐	☐	☐
Smartphone-koppelbares **Blutdruckmessgerät**	☐	☐	☐	☐	☐
**Schlafsensor**, der unter die Matratze gelegt wird			☐	☐	☐

4.2. **Sind Sie mit den folgenden Aussagen betreffend Ihrer Einstellung einverstanden?** (A)

Wählen Sie bitte die auf Sie zutreffende Aussage.

	**Überhaupt nicht einverstanden**	**Nicht einverstanden**	**Neutral**	**Einverstanden**	**Vollständig einverstanden**
Ich denke, dass die Nutzung einer **Smartwatch** in der Schwangerschaftsvorsorge eine gute Idee ist.	☐	☐	☐	☐	☐
Ich denke, dass die häusliche Nutzung eines Smartphone-koppelbaren **Kontraktionszählers** in der Schwangerschaftsvorsorge eine gute Idee ist.	☐	☐	☐	☐	☐
Ich denke, dass die häusliche Nutzung eines Smartphone-koppelbaren **Blutdruckmessgeräts** in der Schwangerschaftsvorsorge eine gute Idee ist.	☐	☐	☐	☐	☐
Ich denke, dass die Nutzung eines **Schlafsensors** in der Schwangerschaftsvorsorge eine gute Idee ist.	☐	☐	☐	☐	☐

4.3. **Werden Sie in Zukunft die unten genannten Geräte in der Schwangerschaftsvorsorge benutzen, wenn Sie diese Möglichkeit bekommen würden?** (BI)

Inwieweit stimmen Sie den untenstehenden Aussagen zu?

	Überhaupt nicht einverstanden	Nicht einverstanden	Neutral	Einverstanden	Vollständig einverstanden
Ich beabsichtige, in Zukunft eine **Smartwatch** in der Schwangerschaft einzusetzen.	☐	☐	☐	☐	☐
Ich beabsichtige, in Zukunft ein Smartphone-koppelbares **Kontraktionszähler** zu Hause einzusetzen.	☐	☐	☐	☐	☐
Ich beabsichtige, in Zukunft ein Smartphone-koppelbares **Blutdruckmessgerät** zu Hause einzusetzen.	☐	☐	☐	☐	☐
Ich beabsichtige, in Zukunft einen **Schlafsensor** einzusetzen.	☐	☐	☐	☐	☐

4.4. **Wie nützlich finden Sie eine SMARTWATCH im Rahmen der Schwangerschaftsvorsorge?** (PE)

Inwieweit stimmen Sie den untenstehenden Aussagen zu, auch wenn Sie keine Smartwatch besitzen?

	Überhaupt nicht einverstanden	Nicht einverstanden	Neutral	Einverstanden	Vollständig einverstanden
Ich finde eine Smartwatch für die Schwangerschaftsvorsorge nützlich.	☐	☐	☐	☐	☐
Die Verwendung einer Smartwatch hilft dabei, gesundheitsbezogene Ziele in der Schwangerschaft schneller zu erreichen.	☐	☐	☐	☐	☐
Die Verwendung einer Smartwatch hilft mir bei meiner täglichen Gesundheitskontrolle in der Schwangerschaft.	☐	☐	☐	☐	☐

4.5. **Wie leicht können Sie so ein technisches Gerät wie eine SMARTWATCH bedienen?** (EE)

Inwieweit stimmen Sie den untenstehenden Aussagen zu?

	Überhaupt nicht einverstanden	Nicht einverstanden	Neutral	Einverstanden	Vollständig einverstanden
Die Funktionalitäten eines technischen Gerätes wie eine Smartwatch sind für mich leicht erlernbar.	☐	☐	☐	☐	☐
Technische Geräte wie Smartwatches sind für mich immer einfach zu bedienen.	☐	☐	☐	☐	☐
Ich bin geschickt im Umgang mit technischen Geräten wie Smartwatches.	☐	☐	☐	☐	☐

4.6. **Wie wichtig ist die Meinung von anderen Leuten für Sie?** (SI)

Wählen Sie bitte die auf Sie zutreffende Aussage.

	Überhaupt nicht einverstanden	Nicht einverstanden	Neutral	Einverstanden	Vollständig einverstanden
**Freunde und Angehörigen** könnten mich dazu bringen, eine Smartwatch für die Schwangerschaftsvorsorge zu verwenden.	☐	☐	☐	☐	☐
**Hebammen und Ärzte/Ärztinnen** könnten mich dazu bringen, eine Smartwatch für die Schwangerschaftsvorsorge zu verwenden.	☐	☐	☐	☐	☐

4.7. **Sind Sie mit den folgenden Aussagen betreffend Ihrem Gesundheitsbewusstsein einverstanden?** (HC)

Wählen Sie bitte die auf Sie zutreffende Aussage.

	Überhaupt nicht einverstanden	Nicht einverstanden	Neutral	Einverstanden	Vollständig einverstanden
Ich denke viel über meine Gesundheit nach.	☐	☐	☐	☐	☐
Normalerweise kenne ich meinen Gesundheitszustand.	☐	☐	☐	☐	☐
Ich achte auf Veränderungen in meiner Gesundheit.	☐	☐	☐	☐	☐
Ich übernehme Verantwortung für meine Gesundheit.	☐	☐	☐	☐	☐

4.8. **Ihre persönlichen Gesundheitsdaten könnten von Smartwatch-Anbietern verwendet werden.** (PPR)

Inwieweit stimmen Sie den untenstehenden Aussagen zu?

	Überhaupt nicht einverstanden	Nicht einverstanden	Neutral	Einverstanden	Vollständig einverstanden
Es ist riskant, meine persönlichen Gesundheitsinformationen den Anderen offenzulegen.	☐	☐	☐	☐	☐
Ich denke, dass das Offenlegen meiner Gesundheitsinformationen mir schaden würde.	☐	☐	☐	☐	☐
Es bestünde zu viel Unsicherheit, wenn ich meine Gesundheitsinformation weitergeben würde.	☐	☐	☐	☐	☐

5. TRI 2.0

Mit Hilfe des folgenden Fragebogens möchten wir verstehen, wie Technologien Ihr Leben beeinflussen.

Inwieweit stimmen Sie den untenstehenden Aussagen zu?

	Überhaupt nicht einverstanden	Nicht einverstanden	Neutral	Einverstanden	Vollständig einverstanden	
Neue Technologien ermöglichen mehr Freiheiten & verbessern die Mobilität der Menschen.	☐	☐	☐	☐	☐	[OPT2]
Neue Technologien erhöhen die Produktivität in meinem persönlichen Leben, d.h. man spart Zeit im Alltag.	☐	☐	☐	☐	☐	[OPT4]
Andere Personen schätzen meine Meinung, wenn es um neue Technologien geht.	☐	☐	☐	☐	☐	[INN1]
Im Allgemeinen bin ich der Erste aus meinem Freundeskreis, der neue Technologien nutzt, wenn sie auf den Markt kommen.	☐	☐	☐	☐	☐	[INN2]
Ich halte mich bzgl. der neuesten technologischen Entwicklungen innerhalb meines Interessensgebiets auf dem Laufenden.	☐	☐	☐	☐	☐	[INN4]
Telefon-Hotlines für technischen Support sind nicht hilfreich, da sie Dinge nicht so erklären, dass ich es verstehen kann.	☐	☐	☐	☐	☐	[DIS2]
Manchmal denke ich, dass technische Geräte nicht für den Gebrauch durch „normale“ Leute gemacht sind.	☐	☐	☐	☐	☐	[DIS3]
Menschen sind zu abhängig von neuen Technologien, die Dinge für sie erledigen.	☐	☐	☐	☐	☐	[INS1]

	Überhaupt nicht einverstanden	Nicht einverstanden	Neutral	Einverstanden	Vollständig einverstanden	
Zu viel Technik lenkt die Menschen so sehr ab, dass es schädlich ist.	☐	☐	☐	☐	☐	[INS2]
Technik verringert die Qualität von Beziehungen durch weniger persönliche Interaktion.	☐	☐	☐	☐	☐	[INS3]

6. PRAQ-R2:

Im Folgenden finden Sie Fragen zu Befürchtungen bezüglich der Geburt Ihres Kindes.

Bitte kreuzen Sie an, wie gut die folgenden Aussagen auf Ihre Situation zutreffen.

Bitte überlegen Sie bei jeder Frage gut, aber nicht zu lange und bearbeiten Sie alle Fragen.

Lassen Sie bitte keine Frage aus.

	Gar nicht relevant	Kaum relevant	Teilweise relevant	Ziemlich relevant	Sehr relevant	
Ich mache mir Sorgen wegen der Schmerzen durch die Wehen und während der Entbindung.	☐	☐	☐	☐	☐	1
Ich habe Angst vor der Entbindung.	☐	☐	☐	☐	☐	2
Ich mache mir Sorgen darum, mich während der Wehen nicht unter Kontrolle zu haben und befürchte, dass ich schreien werde.	☐	☐	☐	☐	☐	3
Ich habe Angst davor, dass das Baby geistig beeinträchtigt sein oder unter einer Gehirnschädigung leiden könnte.	☐	☐	☐	☐	☐	4
Ich habe Angst, dass unser Baby tot geboren wird oder kurz nach der Entbindung stirbt.	☐	☐	☐	☐	☐	5
Ich habe Angst, dass das Baby körperlich beeinträchtigt sein könnte oder fürchte, dass etwas körperlich mit dem Baby nicht stimmt.	☐	☐	☐	☐	☐	6

	Gar nicht relevant	Kaum relevant	Teilweise relevant	Ziemlich relevant	Sehr relevant	
Ich denke manchmal, dass unser Kind bei schlechter Gesundheit oder anfällig für Krankheiten sein wird.	☐	☐	☐	☐	☐	7
Ich bin über die Tatsache besorgt, dass ich nach der Schwangerschaft meine Figur nicht zurückbekommen werde.	☐	☐	☐	☐	☐	8
Ich mache mir Sorgen, dass ich ein unattraktives Erscheinungsbild habe.	☐	☐	☐	☐	☐	9
Ich mache mir über meine hohe Gewichtszunahme Sorgen.	☐	☐	☐	☐	☐	10

Herzlichen Dank für Ihre Geduld und Ihre wertvolle Teilnahme!

Gefördert durch:

Bundesministerium für Gesundheit

aufgrund eines Beschlusses des Deutschen Bundestages
