# Supplementary material for: Using smart devices for prenatal care: Assessing the willingness among women with pregnancy-related anxiety
Source: Digit Health. 2026 Jan 27;12:20552076251406652. doi: 10.1177/20552076251406652 (PMC12847662; doi:10.1177/20552076251406652)
Supplement: sj-docx-2-dhj-10.1177_20552076251406652 - Supplemental material for Using smart devices for prenatal care: Assessing the willingness among women with pregnancy-related anxiety [file sj-docx-2-dhj-10.1177_20552076251406652.docx]

**Supplementary Material 2**


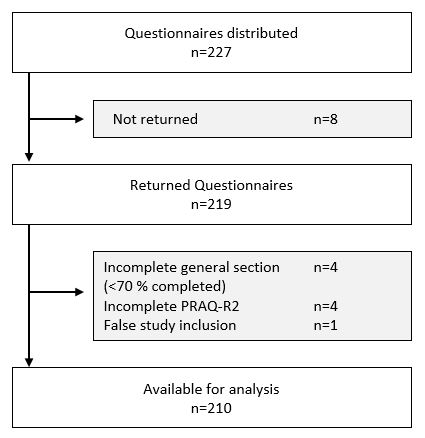


Supplementary Figure 1. Flow chart visualising the questionnaires available for analysis.
