# Supplementary material for: Using smart devices for prenatal care: Assessing the willingness among women with pregnancy-related anxiety
Source: Digit Health. 2026 Jan 27;12:20552076251406652. doi: 10.1177/20552076251406652 (PMC12847662; doi:10.1177/20552076251406652)
Supplement: sj-docx-3-dhj-10.1177_20552076251406652 - Supplemental material for Using smart devices for prenatal care: Assessing the willingness among women with pregnancy-related anxiety [file sj-docx-3-dhj-10.1177_20552076251406652.docx]

**Supplementary Material 3**

| **PRAQ-R2** | **n** | **Minimum Score** | **Maximum Score** | **Mean (SD)** | **Median (IQR)** |
| --- | --- | --- | --- | --- | --- |
| **FoGB** |  |  |  |  |  |
| Low | 144 | 3 | 10 | 6.60 (2.00) | 6.50 (5.00-8.00) |
| High | 66 | 11 | 15 | 12.52 (1.36) | 12.00 (11.00-14.00) |
| Total | 210 | 3 | 15 | 8.46 (3.30) | 8.00 (6.00-11.00) |
| **WaHC** |  |  |  |  |  |
| Low | 152 | 4 | 14 | 9.11 (3.10) | 9.00 (7.00-12.00) |
| High | 58 | 15 | 20 | 17.19 (1.80) | 17.00 (16.00-19.00) |
| Total | 210 | 4 | 20 | 11.34 (4.58) | 11.00 (8.00-15.00) |
| **CoA** |  |  |  |  |  |
| Low | 187 | 3 | 10 | 5.60 (2.21) | 6.00 (3.00-7.00) |
| High | 23 | 11 | 15 | 12.04 (1.33) | 12.00 (11.00-13.00) |
| Total | 210 | 3 | 15 | 6.31 (2.93) | 6.00 (4.00-8.00) |
| **PrA** |  |  |  |  |  |
| Low | 191 | 10 | 37 | 24.54 (6.68) | 25.00 (20.00-30.00) |
| High | 19 | 38 | 48 | 41.89 (3.56) | 42.00 (38.00-45.00) |
| Total | 210 | 10 | 48 | 26.11 (8.15) | 26.00 (21.00-31.00) |

Supplementary Table 1. PRAQ-R2: Pregnancy-related anxiety categories and scores for n=210 participants. Values are expressed as mean (standard deviation) and median (interquartile range).

FoGB: Fear of giving birth; WaHC: Worries about bearing a handycapped child; CoA: Concerns about own appearance; PrA: Pregnancy-related anxiety; SD: standard deviation; IQR: interquartile range.
