# Supplementary material for: Using smart devices for prenatal care: Assessing the willingness among women with pregnancy-related anxiety
Source: Digit Health. 2026 Jan 27;12:20552076251406652. doi: 10.1177/20552076251406652 (PMC12847662; doi:10.1177/20552076251406652)
Supplement: sj-docx-4-dhj-10.1177_20552076251406652 - Supplemental material for Using smart devices for prenatal care: Assessing the willingness among women with pregnancy-related anxiety [file sj-docx-4-dhj-10.1177_20552076251406652.docx]

**Supplementary Material 4**

Supplementary Table 1: Pregnancy-related anxiety group “Fear of giving birth” and associations with participants’ willingness to use a smart device in the context of their prenatal care. Questions were answered on a five-point Likert scale. Analysis of associations was done using Mann-Whitney-U tests to an exact significance level of P=.05. Values are expressed as median (interquartile range) or numbers.

| **Variable** | **High FoGB (n=66),**  **median (IQR)** | **Low FoGB (n=144),**  **median (IQR)** | ***P* value** | **Missing** |
| --- | --- | --- | --- | --- |
| **Frequency of use: “How often would you be willing to use this type of device to better monitor your health during pregnancy and your unborn child?”** |  |  |  |  |
| Smartwatch | 4.00 (3.00-5.00) | 4.00 (2.00-5.00) | .16 | 1 |
| Contraction Counter | 3.00 (2.00-5.00) | 3.00 (2.00-4.00) | .007 | 6 |
| BPM Device | 3.00 (3.00-4.00) | 3.00 (2.00-4.00) | .55 | 7 |
| Sleep Tracker | 3.00 (1.00-3.00) | 3.00 (1.00-3.00) | .26 | 10 |
| **Attitude: “I think that using this type of device in prenatal care is a good idea.** “ |  |  |  |  |
| Smartwatch | 4.00 (3.00-5.00) | 4.00 (3.00-5.00) | .04 | 1 |
| Contraction Counter | 4.00 (4.00-5.00) | 4.00 (3.00-4.00) | <.001 | 1 |
| BPM Device | 4.00 (3.00-5.00) | 4.00 (3.00-5.00) | .40 | 1 |
| Sleep Tracker | 4.00 (3.00-5.00) | 3.00 (3.00-4.00) | .005 | 1 |
| **Intention to use: “Will you use this type of device for prenatal care if you were given the opportunity?**“ |  |  |  |  |
| Smartwatch | 4.00 (3.00-5.00) | 4.00 (3.00-4.00) | .02 |  |
| Contraction Counter | 4.00 (3.00-5.00) | 3.00 (2.00-4.00) | .004 |  |
| BPM Device | 4.00 (3.00-5.00) | 3.00 (2.00-4.00) | .03 |  |
| Sleep Tracker | 3.00 (3.00-4.00) | 3.00 (2.00-4.00) | .01 |  |

FoGB: Fear of giving birth; IQR: interquartile range; n: number

Supplementary Table 2: Pregnancy-related anxiety group “Worries about bearing a handicapped child” and associations with participants’ willingness to use a smart device in the context of their prenatal care. Questions were answered on a five-point Likert scale. Analysis of associations was done using Mann-Whitney-U tests to an exact significance level of P=.05. Values are expressed as median (interquartile range) or numbers.

| **Variable** | **High WaHC (n=58),**  **median (IQR)** | **Low WaHC (n=152),**  **median (IQR)** | ***P* value** | **Missing** |
| --- | --- | --- | --- | --- |
| **Frequency of use: “How often would you be willing to use this type of device to better monitor your health during pregnancy and your unborn child?”** |  |  |  |  |
| Smartwatch | 5.00 (3.00-5.00) | 4.00 (2.00-5.00) | .02 | 1 |
| Contraction Counter | 4.00 (3.00-5.00) | 3.00 (2.00-4.00) | <.001 | 6 |
| BPM Device | 3.00 (2.00-4.00) | 3.00 (2.00-4.00) | .04 | 7 |
| Sleep Tracker | 3.00 (2.00-3.00) | 2.00 (1.00-3.00) | .03 | 10 |
| **Attitude: “I think that using this type of device in prenatal care is a good idea.** “ |  |  |  |  |
| Smartwatch | 4.00 (3.00-5.00) | 4.00 (3.00-5.00) | .57 | 1 |
| Contraction Counter | 4.00 (3.00-5.00) | 4.00 (3.00-4.00) | .03 | 1 |
| BPM Device | 4.00 (3.00-5.00) | 4.00 (3.00-4.00) | .20 | 1 |
| Sleep Tracker | 4.00 (3.00-5.00) | 3.00 (3.00-4.00) | .01 | 1 |
| **Intention to use: “Will you use this type of device for prenatal care if you were given the opportunity?**“ |  |  |  |  |
| Smartwatch | 4.00 (3.00-5.00) | 4.00 (3.00-4.00) | .25 |  |
| Contraction Counter | 4.00 (3.00-5.00) | 3.50 (3.00-4.00) | .18 |  |
| BPM Device | 4.00 (3.00-5.00) | 3.00 (3.00-4.00) | .11 |  |
| Sleep Tracker | 3.00 (3.00-4.00) | 3.00 (2.00-4.00) | .15 |  |

IQR: interquartile range; n: number; WaHC: worries about bearing a handicapped child;

Supplementary Table 3: Pregnancy-related anxiety group “Worries about bearing a handicapped child” and associations with participants’ willingness to use a smart device in the context of their prenatal care. Questions were answered on a five-point Likert scale. Analysis of associations was done using Mann-Whitney-U tests to an exact significance level of P=.05. Values are expressed as median (interquartile range) or numbers.

| **Variable** | **High CoA (n=23),**  **median (IQR)** | **Low CoA (n=187),**  **median (IQR)** | ***P* value** | **Missing** |
| --- | --- | --- | --- | --- |
| **Frequency of use: “How often would you be willing to use this type of device to better monitor your health during pregnancy and your unborn child?”** |  |  |  |  |
| Smartwatch | 3.00 (2.00-5.00) | 4.00 (3.00-5.00) | .56 | 1 |
| Contraction Counter | 3.00 (2.00-4.00) | 3.00 (2.00-4.00) | .65 | 6 |
| BPM Device | 3.00 (2.00-4.00) | 3.00 (4.00-2.00) | .52 | 7 |
| Sleep Tracker | 3.00 (1.00-3.00) | 3.00 (1.00-3.00) | .57 | 10 |
| **Attitude: “I think that using this type of device in prenatal care is a good idea.** “ |  |  |  |  |
| Smartwatch | 4.00 (3.00-5.00) | 4.00 (3.00-5.00) | .83 | 1 |
| Contraction Counter | 4.00 (3.00-5.00) | 4.00 (3.00-4.00) | .08 | 1 |
| BPM Device | 4.00 (3.00-5.00) | 4.00 (3.00-5.00) | .33 | 1 |
| Sleep Tracker | 3.00 (2.00-5.00) | 3.00 (3.00-4.00) | .95 | 1 |
| **Intention to use: “Will you use this type of device for prenatal care if you were given the opportunity?**“ |  |  |  |  |
| Smartwatch | 4.00 (3.00-5.00) | 4.00 (3.00-4.00) | .15 |  |
| Contraction Counter | 4.00 (3.00-5.00) | 3.00 (3.00-4.00) | .06 |  |
| BPM Device | 4.00 (3.00-5.00) | 4.00 (3.00-4.00) | .15 |  |
| Sleep Tracker | 3.00 (2.00-5.00) | 3.00 (2.00-4.00) | .53 |  |

CoA: Concerns about own appearance; IQR: interquartile range; n: number
