# Supplementary material for: Using smart devices for prenatal care: Assessing the willingness among women with pregnancy-related anxiety
Source: Digit Health. 2026 Jan 27;12:20552076251406652. doi: 10.1177/20552076251406652 (PMC12847662; doi:10.1177/20552076251406652)
Supplement: sj-docx-5-dhj-10.1177_20552076251406652 - Supplemental material for Using smart devices for prenatal care: Assessing the willingness among women with pregnancy-related anxiety [file sj-docx-5-dhj-10.1177_20552076251406652.docx]

**Supplementary Material 5**

Supplementary Table 1: Pregnancy related anxiety group “Fear of giving birth” and associations with the perceived usefulness (performance expectancy) of a smartwatch’s use in the context of prenatal care. Questions were answered on a five-point Likert scale. Analysis of associations was done using Mann-Whitney-U tests to an exact significance level of P=.05. Values are expressed as mean (standard deviation) or numbers.

| **Variable** | **High FoGB (n=66),**  **median (IQR))** | **Low FoGB (n=144),**  **median (IQR)** | ***P* value** |
| --- | --- | --- | --- |
| I think it could be useful. | 4.00 (4.00-5.00) | 4.00 (3.00-4.00) | .02 |
| It could help to accomplish health-related aims in prenatal care more quickly. | 4.00 (3.00-5.00) | 4.00 (3.00-4.00) | .003 |
| It could help me with my daily health checks during pregnancy. | 4.00 (3.00-5.00) | 4.00 (3.00-4.00) | .08 |
| Performance expectancy | 12.00 (10.00-15.00) | 12.00 (9.25-13.00) | .02 |

FoGB: Fear of giving birth; IQR: interquartile range; n: number

Supplementary Table 2: Pregnancy related anxiety group “Worries about bearing a handicapped child” and associations with the perceived usefulness (performance expectancy) of a smartwatch’s use in the context of prenatal care. Questions were answered on a five-point Likert scale. Analysis of associations was done using Mann-Whitney-U tests to an exact significance level of P=.05. Values are expressed as mean (standard deviation) or numbers.

| **Variable** | **High WaHC (n=58),**  **median (IQR)** | **Low WaHC (n=152),**  **median (IQR)** | ***P* value** |
| --- | --- | --- | --- |
| I think it could be useful. | 4.00 (3.00-5.00) | 4.00 (3.00-4.75) | .59 |
| It could help to accomplish health-related aims in prenatal care more quickly. | 4.00 (3.00-5.00) | 4.00 (3.00-4.00) | .26 |
| It could help me with my daily health checks during pregnancy. | 4.00 (3.00-5.00) | 4.00 (3.00-4.00) | .24 |
| Performance expectancy | 12.00 (10.00-15.00) | 12.00 (10.00-13.00) | .33 |

IQR: interquartile range; n: number; WaHC: worries about bearing a handicapped child

Supplementary Table 3: Pregnancy related anxiety group “Concerns about own appearance” and associations with the perceived usefulness (performance expectancy) of a smartwatch’s use in the context of prenatal care. Questions were answered on a five-point Likert scale. Analysis of associations was done using Mann-Whitney-U tests to an exact significance level of P=.05. Values are expressed as mean (standard deviation) or numbers.

| **Variable** | **High CoA (n=23),**  **median (IQR)** | **Low CoA (n=187),**  **median (IQR)** | ***P* value** |
| --- | --- | --- | --- |
| I think it could be useful. | 4.00 (3.00-5.00) | 4.00 (3.00-5.00) | .38 |
| It could help to accomplish health-related aims in prenatal care more quickly. | 4.00 (4.00-5.00) | 4.00 (3.00-4.00) | .03 |
| It could help me with my daily health checks during pregnancy. | 4.00 (4.00-5.00) | 4.00 (3.00-4.00) | .04 |
| Performance expectancy | 12.00 (11.00-15.00) | 12.00 (10.00-13.00) | .07 |

CoA: Concerns about own appearance; IQR: interquartile range; n: number
